# Baseline assessment of WHO’s target for both availability and affordability of essential medicines to treat non-communicable diseases

**DOI:** 10.1371/journal.pone.0171284

**Published:** 2017-02-07

**Authors:** Margaret Ewen, Marjolein Zweekhorst, Barbara Regeer, Richard Laing

**Affiliations:** 1 Health Action International, Amsterdam, Netherlands; 2 Athena Institute, VU University, Amsterdam, Netherlands; 3 Boston University School of Public Health, Boston, MA, United States of America; 4 University of Western Cape, Cape Town, South Africa; University of Rijeka, CROATIA

## Abstract

**Background:**

WHO has set a voluntary target of 80% availability of affordable essential medicines, including generics, to treat major non-communicable diseases (NCDs), in the public and private sectors of countries by 2025. We undertook a secondary analysis of data from 30 surveys in low- and middle-income countries, conducted from 2008–2015 using the World Health Organization (WHO)/Health Action International (HAI) medicine availability and price survey methodology, to establish a baseline for this target.

**Methods:**

Data for 49 medicines (lowest priced generics and originator brands) to treat cardiovascular diseases (CVD), diabetes, chronic obstructive pulmonary diseases (COPD) and central nervous system (CNS) conditions were analysed to determine their availability in healthcare facilities and pharmacies, their affordability for those on low incomes (based on median patient prices of each medicine), and the percentage of medicines that were both available and affordable. Affordability was expressed as the number of days’ wages of the lowest-paid unskilled government worker needed to purchase 30 days’ supply using standard treatment regimens. Paying more than 1 days’ wages was considered unaffordable.

**Findings:**

In low-income countries, 15.2% and 18.9% of lowest-priced generics met WHO’s target in the public and private sectors, respectively, and 2.6% and 5.2% of originator brands. In lower-middle income countries, 23.8% and 23.2% of lowest priced generics, and 0.8% and 1.4% of originator brands, met the target in the public and private sectors, respectively. In upper-middle income countries, the situation was better for generics but still suboptimal as 36.0% and 39.4% met the target in public and private sectors, respectively. For originator brands in upper-middle income countries, none reached the target in the public sector and 13.7% in the private sector. Across the therapeutic groups for lowest priced generics, CVD medicines in low-income countries (11.9%), and CNS medicines in lower-middle (10.2%) and upper-middle income countries (33.3%), were least available and affordable in the public sector. In the private sector for lowest priced generics, CNS medicines were least available and affordable in all three country income groups (11.4%, 5.8% and 29.3% in low-, lower-middle and upper-middle income countries respectively).

**Interpretation:**

This data, which can act as a baseline for the WHO target, shows low availability and/or poor affordability is resulting in few essential NCD medicines meeting the target in low- and middle-income countries. In the era of Sustainable Development Goals, and as countries work to achieve Universal Health Coverage, increased commitments are needed by governments to improve the situation through the development of evidence-informed, nationally-contextualised interventions, with regular monitoring of NCD medicine availability, patient prices and affordability.

## Introduction

Attention is growing on preventing and treating non-communicable diseases (NCDs) which, according to the World Health Organization (WHO) are now the world’s biggest killers [1]. Over 36 million people die annually (63% of global deaths) from NCDs, mainly cardiovascular diseases, cancer, chronic respiratory diseases and diabetes. Of these, 80% occur in low- and middle-income countries [1]. Complications from hypertension accounts for 9.4 million deaths worldwide annually [2]. Diabetes is also of concern. By 2035, an estimated 592 million people will have diabetes, a 55% increase over the 2013 to 2035 period [3].

Following the Political Declaration on NCDs, adopted by the UN General Assembly in 2011, WHO published its Global Action Plan for the Prevention and Control of NCDs 2013–2020 (GAP), which was endorsed by the World Health Assembly in 2013 [1,4,5]. Included are six objectives, one of which is to strengthen health systems to improve prevention, detection, treatment and management of people with or at high risk for cardiovascular diseases, diabetes, chronic respiratory diseases, cancer and other NCDs. This objective includes improving patient access to affordable medicines to treat NCDs.

NCDs are different to acute diseases in that most require uninterrupted, life-long treatment. To help achieve this, NCD medicines must be both available in facilities when needed and affordable, especially for those on low-incomes. Using savings, borrowing money or selling assets to pay for healthcare, a common occurrence in low-income countries [6], is not a sustainable option for people with NCDs. The WHO recognised this and included in the GAP a voluntary target of 80% availability of affordable basic technologies and essential medicines, including generics, required to treat major NCDs in both public and private facilities by 2025 [5].

In the GAP, medicine affordability was not defined, which is not surprising as it is not straightforward. However, the measure used by WHO and HAI in their methodology to measure medicine prices, availability and affordability [7] (number of days’ wages needed by the lowest-paid unskilled government worker to purchase standard treatments) is widely accepted and used, and clearly shows the reality for those on low wages who must pay out-of-pocket for medicines [8–14]. But while being easy to apply and understand, it may overestimate medicine affordability because in many countries a substantial proportion of the population earn less than this government worker [7,8]. Niëns et al. proposed a different metric for expressing medicine affordability but it has not been widely used [15].

National and sub-national surveys using the WHO/HAI methodology have shown poor availability (particularly in the public sector) and poor affordability of medicines to treat a range of NCDs including cardiovascular diseases, diabetes, psychiatric disorders, asthma and epilepsy [8–12,16]. In 2007 Mendis et al. assessed the availability and affordability of medicines to treat four NCDs in six low- and middle-income countries using an adaptation of the WHO/HAI methodology [12]. They found generic availability did not exceed 7.5% in the public sector in Bangladesh, Malawi, Nepal and Pakistan, and standard treatments often required 5 or more days’ wages when purchased in the private sector. More recently, NCD surveys using the WHO/HAI methodology have been undertaken in several countries in the Middle East. In Lebanon [13], the availability of generics was low in primary healthcare centres (43.3%) where medicines are dispensed free-of-charge, but higher in private pharmacies (77.9%). Standard treatments with lowest priced generics were generally affordable in the private sector, except for some medicines to treat neuroleptic disorders. Less affordable were originator brands and/or when a patient needs multiple medicines. Similar findings on treatment affordability were found in Egypt where treating a person with co-morbidities, such as diabetes, hypertension and hypercholesterolaemia would be largely unaffordable for those on low wages [14]. Cameron et al. found the availability of medicines in developing countries was suboptimal in the public and private sectors, with generics to treat NCDs significantly less available than generics for acute conditions [17].

Studies to date using WHO/HAI data have considered availability and affordability separately. But, as noted in the GAP, to improve access medicines must be both available in facilities and affordable for all who need them. Therefore, in this study, we looked at the combined availability and affordability of medicines to treat four major NCDs (cardiovascular disease (CVD), diabetes, chronic obstructive pulmonary diseases (COPD), and central nervous system conditions (CNS)) to ascertain the extent to which they met WHO’s target.

## Methods

### WHO/HAI medicine price, availability and affordability surveys

The WHO/HAI methodology is a facility-based survey of the availability and patient price of 50 medicines to treat communicable and non-communicable diseases, in a minimum of six geographic or administrative areas in a sample of medicine outlets in the public sector, private sector, and other sectors [7]. Data are also collected on government procurement prices and price components in the pharmaceutical supply chain (mark-ups, taxes etc.). Most surveys are undertaken at the national level, although subnational surveys are recommended in large countries.

Data is collected from five medicine outlets per sector per survey area. The selection of the outlets uses a multistage clustered approach, as described by Cameron et al [8]. Survey medicines include a global core list (14), all strength- and dosage-form specific, commonly used to treat a range of conditions that cause substantial morbidity and mortality (e.g. hypertension, diabetes, respiratory tract infection). Supplementary medicines (36) of local importance are also surveyed. For each medicine, data are collected for the originator brand, and the lowest-priced generic equivalent found in each outlet.

Data is analysed by sector. Availability, reported as the percentage of outlets where the medicine was in stock on the day of data collection, takes into account the level of outlet in the public sector that is permitted to stock each medicine. Prices are expressed as medians in local currency, and as a ratio to median supplier prices (or the median buyer price when no supplier price is given) listed in Management Sciences for Health’s (MSH) *International Drug Price Indicator Guide*. A minimum of four patient prices per medicine per sector are needed for inclusion in the analysis.

Affordability is based on median patient prices (originator brand and lowest priced generics) of each medicine in local currency for a standard treatment regimen, and expressed as the number of days’ wages needed by the lowest-paid unskilled government worker to purchase 30 days’ supply of the medicine to treat NCDs, and 7 days’ supply for medicines to treat communicable diseases. Where a medicine is not available or there are less than four price points, affordability is not assessed.

### Methodology of the secondary analysis

#### Survey selection

Data for the secondary analysis were obtained from 30 surveys undertaken in low-income and middle-income countries from 2008 to early 2015 using the WHO/HAI methodology, and published on the WHO/HAI price database [18]. Based on 2014 World Bank income groups, 10 surveys were conducted in low-income countries, 12 in lower-middle income countries, and 8 in upper-middle-income countries. High-income countries were excluded as only 3 had been surveyed in this period. Table 1 lists the surveys in the analysis, the number of outlets sampled per survey, plus the daily wage of the lowest-paid unskilled government worker per country.

**Table 1:** Surveys in secondary analysis of NCD medicine availability and affordability.

Country (survey year)	World Bank Income Group (2014)	Number of medicine outlets surveyed^a^	Daily wage lowest-paid unskilled government worker in local currency (in USD^b^)
Afghanistan (2011)	Low	116	150 Afghani ($3.03)
African country (2008)^c^	Low	48	$1.32
African country (2013)^c^	Low	94	$2.33
Bolivia (2008)	Lower-middle	60	19.25 Boliviano **(**$2.72)
Burkina Faso (2009)	Low	65	1023 FCFA ($2.17)
Burundi (2013)	Low	50	2692 Burundi Franc ($1.78)
Brazil, Rio Grande de Sol (2008)	Upper-middle	52	12.73 Real ($5.49)
China, Shaanxi Province (2014)	Upper-middle	140	37.3333 Chinese Yuan Renminbi ($6.06)
Colombia (2008)	Upper-middle	89	15383 Colombian Peso ($6.44)
Ecuador (2008)	Upper-middle	60	$6.67
Ethiopia (2013)	Low	64	14 Birr ($0.75)
Haiti (2011)	Low	89	200 Gourde ($5.04)
India, NCT, Delhi (2011)	Lower-middle	113	247 Indian Rupee ($5.53)
Indonesia (2010)	Lower-middle	144	36500 Rupiah ($3.98)
Iran (2014)	Upper-middle	60	270000 Rial ($10.78)
Kyrgyzstan (2015)	Lower-middle	35^d^	46.19 Som ($0.71)
Lao PDR (2013)	Lower-middle	60	20867 Kip ($2.62)
Latin American country (2009)^c^	Lower-middle	70	$6.92
Lebanon (2013)	Upper-middle	60	22500 Lebanese Pound ($14.93)
Mauritius (2008)	Upper-middle	60	215 Mauritius Rupee($8.27)
Mexico, Mexico City (2009)	Upper-middle	28	57.64 Mexican Peso ($4.48)
Moldova (2011)	Lower-middle	100	20 Lei ($1.69)
Mongolia (2012)	Lower-middle	66	6686 Tugrik ($4.79)
Nicaragua (2008)	Lower-middle	61	60.03 Cordoba ($3.06)
Sao Tomé et Principe (2008)	Lower-middle	41	18150 Dobra ($1.25)
Sudan (2013)	Lower-middle	71	12 Sudanese Pound ($1.85)
Tanzania (2012)^e^	Low	73	5667 Tanzanian Shilling ($3.65)
Tajikistan (2013)	Low	60	6.67 Somoni ($1.40)
Uganda (2015) ^e^	Low	66	5200 Ugandan Shilling($2.03)
Ukraine (2012)	Lower-middle	70	41.5915 Hryvnia ($5.21)
**Total: 30 countries**		**2161 outlets**	**Mean: $4.23 Range: $0.71-$14.93**

^a^Public and private sector

^b^Based on the exchange rate used in the survey

^c^Permission not given to identify country

^d^Private sector only

^e^Only lowest priced generics were surveyed

WHO World Health Organization; USD United States of America dollars.

#### Therapeutic group and medicine selection

Four groups of NCDs were included in the analysis i.e. CVD, diabetes, COPD, and CNS conditions i.e. psychoses, anxiety, depression, epilepsy and Parkinson’s Disease. These had the greatest amount of data in the WHO/HAI database. A preliminary analysis identified the most surveyed medicines in each therapeutic group which, when combined, covered at least 80% of data points within the group.

For each survey, only medicines on the National Essential Medicines List (NEML) at the time of the survey were selected for the secondary analysis. For Haiti there was no NEML at the time of the survey, however, it was published the following year so this was used to identify which survey medicines were essential. For Lebanon, an updated NEML was published a few months after this survey so that was used to identify the essential medicines. In total, 18 medicines to treat CVD, 7 to treat diabetes, 9 to treat COPD, and 15 CNS medicines (all strength- and dosage-form specific) were included in the secondary analysis (Table 2).

**Table 2:** NCD medicines in secondary analysis of availability and affordability.

Medicine, strength and dosage form	Percentage of surveys with medicine(n = no. of surveys)	No. units for affordability analysis(30 days’ supply)
***Medicines for cardiovascular diseases***		
Furosemide 40mg cap/tab	83.3% (n = 25)	30
Atenolol 50mg cap/tab*	73.3% (n = 22)	30
Enalapril 5mg,10mg and 20mg cap/tab*	70.0% (n = 21)	30
Simvastatin 20mg cap/tab*	66.7% (n = 20)	30
Captopril 25mg cap/tab*	63.3% (n = 19)	60
Amlodipine 5mg cap/tab	46.7% (n = 14)	30
Hydrochlorothiazide 25mg cap/tab*	46.7% (n = 14)	30
Atorvastatin 10mg and 20mg cap/tab*	30.0% (n = 9)	30
Nifedipine Retard 20mg tab*	30.0% (n = 9)	60
Digoxin 0.25mg cap/tab	30.0% (n = 9)	30
Acetylsalicyclic acid 100mg cap/tab*	26.7% (n = 8)	30
Losartan 50mg cap/tab	20.0% (n = 6)	30
Propranolol 40mg cap/tab*	16.7% (n = 5)	120
Isosorbide dinitrate 10mg cap/tab	13.3% (n = 4)	180
Lisinopril 10mg cap/tab*	10.0% (n = 3)	30
***Medicines for diabetes***		
Glibenclamide 5mg cap/tab	93.3% (n = 28)	60
Metformin 500mg and 850mg cap/tab*	76.7% (n = 23)	500mg 90; 850mg 60
Insulin human, soluble, isophane and 30/70, 100IU/ml vial*	30.0% (n = 9)	10ml
Gliclazide 80mg cap/tab	13.3% (n = 4)	30
***Medicines for chronic obstructive pulmonary diseases (COPD)***		
Salbutamol 100mcg/dose inhaler*	96.7% (n = 29)	200 doses
Beclometasone 50mcg/dose, 100mcg/dose and 250mcg/dose inhaler*	56.7% (n = 17)	200 doses
Budesonide 100mcg/dose and 200mcg/dose inhaler*	10.0% (n = 3)	200 doses
Ipratropium 20mcg/dose inhaler*	3.3% (n = 1)	200 doses
Salbutamol 2mg and 4mg cap/tab	13.3% (n = 4)	2mg 180; 4mg 90
***Medicines for central nervous system (CNS) conditions*: *antipsychotics*, *medicines for anxiety*, *depression*, *epilepsy and Parkinson’s Disease***		
Amitriptyline 25mg cap/tab	96.7% (n = 29)	90
Diazepam 5mg and 10mg cap/tab	83.3% (n = 25)	7
Carbamazepine 200mg cap/tab	76.7% (n = 23)	150
Fluoxetine 20mg cap/tab	53.3% (n = 16)	30
Phenytoin 100mg cap/tab	43.3% (n = 13)	90
Clonazepam 2mg cap/tab	23.3% (n = 7)	120
Phenobarbital 100mg and 30mg cap/tab	16.7% (n = 5)	100mg 30;30mg 90
Clozapine 100mg cap/tab	13.3% (n = 4)	90
Sodium valproate 200mg and valproic acid 150mg cap/tab	10.0% (n = 3)	200mg 150; 150mg 200
Risperidone 2mg cap/tab	6.7% (n = 2)	60
Imipramine 25mg cap/tab	6.7% (n = 2)	120
Levodopa+carbidopa 25+250mg cap/tab	6.7% (n = 2)	120

* Belong to the classes of NCD medicines in the GAP report considered necessary to provide basic cost-effective primary healthcare

GAP lists medicines needed to provide basic, cost-effective primary healthcare i.e. 6 CVD medicines (aspirin, a statin, a thiazide diuretic, a beta-blocker, an ACE inhibitor, and a long-acting calcium channel blocker), two products to treat diabetes (metformin and insulin), and two inhalers (bronchodilator and steroid). We used an expanded list (including CNS medicines) in our analysis as they were selected nationally to be surveyed hence considered important.

#### Data analysis

The basis of all analyses was the availability and/or affordability of individual medicines in each survey. Data were not aggregated in the few surveys where two strengths or two dosage forms of the same medicine were surveyed, or by country.

Two surveys (Tanzania and Uganda) did not collect data for originator brands of the survey medicines. In some other surveys, data on originator brands or generics of some medicines were not collected as marketing authorisation had not been granted in the country. In these cases, the originator brand or lowest priced generic was excluded from the analyses. As well, Tanzania and Uganda was excluded from the analysis of availability of any product type.

Data were stratified by World Bank income levels to low-income countries, lower-middle income countries and upper-middle income countries. Within each income group, the median percentage availability across the basket of medicines in each therapeutic group, and across all the medicines, was calculated for originator brands and generics, separately and combined, in the public and private sectors.

The median number of days’ wages to purchase standard treatments was calculated across the basket of medicines in each therapeutic group, and across all medicines. The combined availability and affordability was then determined to identify the percentage of medicines within a therapeutic group, and across all medicines, with 80% or greater availability and requiring 1 days’ wages or less to purchase standard treatments or supplied free-of-charge in the public sector.

Whether low availability was the sole cause for not achieving the WHO target, or poor affordability, or both low availability and poor affordability, was then assessed for each therapeutic group per sector.

All the primary data for each medicine in the analysis is available in the WHO/HAI price database i.e. percentage availability, median patient price in local currency (as well as in US dollars and ratio to the MSH international reference price) and the number of days’ wages needed by the lowest-paid unskilled government worker to purchase 30 days’ supply [18].

## Findings

### Availability

In all three country income groups, the median availability of generics did not exceed 80% for any therapeutic group in the public sector and only for CVD medicines in the private sector of lower-middle income countries (85.8%) and upper-middle income countries (86.7%) as shown in Table 3. In low-income countries, median generic availability (across all medicines) was 40.2% and 59.1% in the public and private sectors, respectively. In the more wealthy countries, overall generic availability was higher at 54.6% and 65.7% (lower-middle) and 56.7% and 76.7% (upper-middle) in the public and private sectors, respectively.

**Table 3:** Median percentage availability by World Bank Income Group.

World Bank Income Group	Therapeutic group	Median % availability					
		Public sector			Private sector		
		Originator brand	Lowest priced generic	Any product	Originator brand	Lowest priced generic	Any product
Low-income countries (n = 10)	Cardiovascular	0% (n = 31)	42.9% (n = 42)	45.0%* (n = 34)	3.3% (n = 30)	68.6% (n = 41)	82.9%* (n = 33)
	Diabetes	0% (n = 14)	51.3% (n = 18)	57.4%* (n = 14)	12.1% (n = 14)	65.2% (n = 18)	69.5%* (n = 14)
	COPD	3.2% (n = 13)	25.8% (n = 17)	29.0%* (n = 13)	20.0% (n = 13)	44.0% (n = 17)	83.3%* (n = 13)
	CNS	0% (n = 20)	44.1% (n = 35)	35.7%* (n = 28)	0% (n = 20)	45.7% (n = 35)	46.4%* (n = 28)
	*All medicines*	0% (n = 78)	40.2% (n = 112)	43.3%* (n = 89)	3.2% (n = 77)	59.1% (n = 111)	66.7%* (n = 88)
Lower-middle income countries (n = 12)	Cardiovascular	0% (n = 52)	74.2% (n = 80)	74.2% (n = 80)	6.7% (n = 62)	85.8% (n = 90)	88.6% (n = 90)
	Diabetes	1.2% (n = 18)	52.9% (n = 24)	59.5% (n = 24)	24.0% (n = 20)	66.2% (n = 26)	71.0% (n = 26)
	COPD	4.1% (n = 18)	51.4% (n = 19)	51.4% (n = 19)	18.6% (n = 20)	59.2% (n = 22)	63.3% (n = 22)
	CNS	0% (n = 36)	34.3% (n = 49)	37.1% (n = 49)	3.3% (n = 39)	37.1% (n = 52)	42.1% (n = 52)
	*All medicines*	0% (n = 124)	54.6% (n = 172)	57.6% (n = 172)	10.0% (n = 141)	65.7% (n = 190)	68.6% (n = 190)
Upper-middle income countries (n = 8)	Cardiovascular	0% (n = 49)	58.4% (n = 58)	60.3% (n = 58)	55.6% (n = 49)	86.7% (n = 55)	93.3% (n = 56)
	Diabetes	0% (n = 17)	61.7% (n = 22)	64.1% (n = 22)	60.0% (n = 17)	71.7% (n = 22)	89.9% (n = 22)
	COPD	0% (n = 14)	64.1% (n = 14)	64.1% (n = 14)	32.6% (n = 14)	59.7% (n = 14)	81.7% (n = 14)
	CNS	0% (n = 37)	46.7% (n = 42)	46.7% (n = 42)	45.8% (n = 37)	66.7% (n = 41)	86.7% (n = 41)
	*All medicines*	0% (n = 117)	56.7% (n = 136)	60.2% (n = 136)	53.3% (n = 117)	76.7% (n = 132)	90.0% (n = 133)

*Excludes data points for Uganda and Tanzania as originator brands were not surveyed; n = number of data points in the analysis

In the public sector, median availability of originator brands across all medicines was 0% in all three country income groups. In the private sector, overall availability of originator brands was 3.2%, 10% and 53.3% in low-, lower-middle and upper-middle income countries, respectively.

In low-income countries, the median availability of any product type (originator brands and generics) across all medicines was 43.3% and 66.7% in the public and private sectors, respectively. In lower-middle income countries, median availability for any product type was 57.6% and 68.6% in the public and private sectors respectively. In upper-middle income countries, median availability for any product type was similar to the less wealthy countries in the public sector (60.2%) but much higher in the private sector (90.0%). For any product type, median availability was less than 80% for all four therapeutic groups in the public sector. In the private sector, availability was 80% or higher for CVD (82.9%) and COPD (83.3%) medicines in low-income countries, CVD (88.6%) in lower-middle income countries, and all four therapeutic groups in upper-middle income countries.

### Affordability

Based on the median number of days’ wages needed to purchase treatments, buying lowest-priced generics in the public sector requires no more than 1 days’ wage in each therapeutic groups, except for COPD and CNS medicines in lower-middle income countries which require 1.4 days’ wages (Table 4). In the private sector, no more than 1 days’ wage was needed in upper-middle income countries for all four therapeutic groups, and CVD medicines in low-income countries.

**Table 4:** Median number of days’ wages needed to purchase standard treatments, by World Bank Income Group.

World Bank Income Group	Therapeutic group	Median days’ wages*			
		Public sector		Private sector	
		Originator brand	Lowest priced generic	Originator brand	Lowest priced generic
Low-income countries (n = 10)	Cardiovascular	1.9 (n = 4)	0.6 (n = 28)	2.9 (n = 11)	0.9 (n = 38)
	Diabetes	2.9 (n = 2)	0.9 (n = 9)	5.3 (n = 6)	1.1 (n = 15)
	COPD	0.9 (n = 2)	0.7 (n = 9)	2.9 (n = 8)	1.3 (n = 14)
	CNS	1.1 (n = 2)	0.4 (n = 15)	1.3 (n = 6)	1.1 (n = 28)
	*All medicines*	1.1 (n = 10)	0.7 (n = 61)	3.1 (n = 31)	1.0 (n = 95)
Lower-middle income countries (n = 12)	Cardiovascular	2.8 (n = 7)	0.7 (n = 58)	3.7 (n = 31)	1.1 (n = 85)
	Diabetes	3.3 (n = 4)	0.6 (n = 16)	3.8 (n = 13)	1.4 (n = 24)
	COPD	2.4 (n = 6)	1.4 (n = 10)	2.5 (n = 13)	1.7 (n = 18)
	CNS	12.0 (n = 4)	1.4 (n = 30)	9.0 (n = 14)	2.3 (n = 40)
	*All medicines*	3.0 (n = 21)	0.9 (n = 114)	3.8 (n = 71)	1.4 (n = 167)
Upper-middle income countries (n = 8)	Cardiovascular	3.5 (n = 6)	0.1 (n = 17)	1.9 (n = 39)	0.3 (n = 55)
	Diabetes	2.1 (n = 2)	0.3 (n = 6)	1.4 (n = 14)	0.5 (n = 22)
	COPD	0.6 (n = 1)	0.3 (n = 3)	1.5 (n = 9)	0.6 (n = 12)
	CNS	-	0.4 (n = 10)	4.0 (n = 28)	0.9 (n = 32)
	*All medicines*	2.8 (n = 9)	0.2 (n = 36)	2.4 (n = 90)	0.5 (n = 121)

*Based on median treatment prices and the daily wage of the lowest paid unskilled government worker. Excludes medicines supplied free-of-charge in the public sector. n = number of data points in the analysis

In all three country groups, originator brands were less affordable than lowest-priced generics in both sectors. Originator brands of CNS medicines were least affordable requiring 12 and 9 days’ wages to purchase 30 days’ supply in the public and private sectors respectively of lower-middle income countries. Note: the analysis for originator brands in the public sector is based on only a few data points per therapeutic group.

### Meeting WHO’s target for availability and affordability

The percentage of essential medicines with 80% or greater availability and requiring no more than 1 days’ wages to purchase treatments (or supplied free-of-charge in the public sector) was low in all three country groupings as shown in Table 5. Overall in low-income countries, 15.2% and 18.9% of lowest priced generics met this availability and affordability target in the public and private sectors, respectively. They ranged from 11.9% (CVD) to 23.5% (COPD) in the public sector, and 11.4% (CNS) to 27.8% (diabetes) in the private sector. The percentage of lowest-priced generics (across all medicines) meeting the target increased as the wealth of the countries increased, although they remained sub-optimal. In lower-middle income countries, 23.8% and 23.2% met the target in the public and private sectors, respectively. Lowest levels were seen for CNS medicines, and highest for CVD, in both sectors. In upper-middle income countries, 36.0% and 39.4% of lowest-priced generics met the target in the public and private sectors, respectively. In the public sector, they ranged from 33.3% (CNS) to 45.5% (diabetes). In the private sector, they ranged from 29.3% (CNS) to 50.9% (CVD).

**Table 5:** Percentage of data points where medicines were both available and affordable, by World Bank Income Group.

World Bank Income Group	Therapeutic group	Medicines available and affordable*			
		Public sector		Private sector	
		Originator brand	Lowest priced generic	Originator brand	Lowest priced generic
Low-income countries (n = 10)	Cardiovascular	3.2% (1/31)	11.9% (5/42)	3.3% (1/30)	22.0% (9/41)
	Diabetes	0.0% (0/14)	16.7% (3/18)	7.1%(1/14)	27.8%(5/18)
	COPD	0.0% (0/13)	23.5% (4/17)	7.7%(1/13)	17.6%(3/17)
	CNS	5.0% (1/20)	14.3% (5/35)	5.0%(1/20)	11.4%(4/35)
	*All medicines for all therapeutic groups*	2.6% (2/78)	15.2% (17/112)	5.2%(4/77)	18.9%(21/111)
Lower-middle income countries (n = 12)	Cardiovascular	0.0% (0/52)	33.8% (27/80)	1.6%(1/62)	36.7%(33/90)
	Diabetes	0.0% (0/18)	20.8% (5/24)	0.0% (0/20)	23.1%(6/26)
	COPD	0.0% (0/18)	21.1% (4/19)	5.0%(1/20)	9.1% (2/22)
	CNS	2.8% (1/36)	10.2% (5/49)	0.0%(0/39)	5.8%(3/52)
	*All medicines for all therapeutic groups*	0.8% (1/124)	23.8% (41/172)	1.4%(2/141)	23.2%(44/190)
Upper-middle income countries (n = 8)	Cardiovascular	0.0% (0/49)	34.5%(20/58)	20.4%(10/49)	50.9%(28/55)
	Diabetes	0.0% (0/17)	45.5% (10/22)	11.8% (2/17)	31.8% (7/22)
	COPD	0.0% (0/14)	35.7% (5/14)	14.3% (2/14)	35.7% (5/14)
	CNS	0.0% (0/37)	33.3% (14/42)	5.4% (2/37)	29.3% (12/41)
	*All medicines for all therapeutic groups*	0.0% (0/117)	36.0% (49/136)	13.7% (16/117)	39.4% (52/132)

*80% or greater availability and requiring 1 days’ wages or less to purchase 30 days’ supply or supplied free-of-charge in the public sector.

Across all medicines, very few originator brands met the target in low-income countries (2.6% and 5.2% in the public and private sectors, respectively) and lower-middle income countries (0.8% and 1.4%). In upper-middle income countries, no originator brands met the target in the public sector, and 13.7% in the private sector.

Where the WHO target was not meet, we identified whether the cause was solely low availability, solely poor affordability, or both low availability and poor affordability (Table 6). In the public sector for lowest-priced generics, the cause was predominantly low availability (78.9%, 59.5% and 98.9% in low-, lower-middle and upper-middle income countries, respectively). The same was found in the public sector for originator brands (93.4%, 85.4% and 94.9% in low-, lower-middle and upper-middle income countries, respectively). These public sector findings were unsurprising as a number of the countries in the analysis provide medicines free-of-charge so affordability for the patient is not relevant. It must also be remembered that when availability is very low, affordability is not calculated. In the private sector, a mix of causes was found. For lowest-priced generics in the private sector of low- and upper-middle income countries, the predominant cause was low availability (48.9% and 63.7% respectively) but in lower-middle income countries the predominant cause was a combination of low availability and poor affordability (43.2%). For originator brands in the private sector of low- and lower-middle income countries, the predominant cause was low availability (68.5% and 58.3% respectively) but in upper-middle income countries the predominant cause was a combination of low availability and poor affordability (50.5%).

**Table 6:** Percentage cause of not meeting the WHO target (poor availability, poor affordability, or both).

Sector	Medicine type	Low availability only	Poor affordability only	Both low availability and poor affordability
*Low-income countries (n = 10)*				
Public	Originator brand	93.4% (71/76)	0% (0/76)	6.7% (5/76)
	Lowest priced generic	78.9% (75/95)	3.2% (3/95)	17.9% (17/95)
Private	Originator brand	68.5% (50/73)	2.7% (2/73)	28.8% (21/73)
	Lowest priced generic	48.9% (44/90)	12.2% (11/90)	38.9% (35/90)
*Lower-middle income countries (n = 12)*				
Public	Originator brand	85.4% (105/123)	0% (0/123)	14.6% (18/123)
	Lowest priced generic	59.5% (78/131)	11.5% (15/131)	29.0% (38/131)
Private	Originator brand	58.3% (81/139)	0% (0/139)	41.7% (58/139)
	Lowest priced generic	35.6% (52/146)	21.2% (31/146)	43.2% (63/146)
*Upper-middle income countries (n = 8)*				
Public	Originator brand	94.9% (111/117)	0% (0/117)	5.1% (6/117)
	Lowest priced generic	98.9% (86/87)	0% (0/87)	1.1% (1/87)
Private	Originator brand	31.7% (32/101)	17.8% (18/101)	50.5% (51/101)
	Lowest priced generic	63.7% (51/80)	16.3% (13/80)	20.0% (16/80)

**Fig 1:**
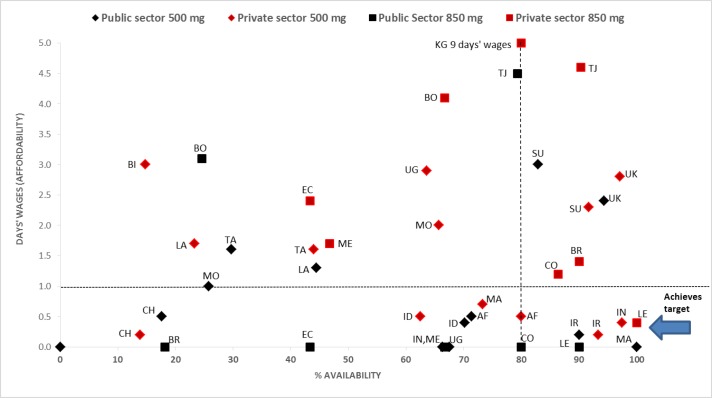
Availability and affordability of metformin 500mg and 850mg tabs, lowest priced generics, by sector and country. AF Afghanistan, BI Burundi, BO Bolivia, BR Brazil Rio Grande de Sol, CH China Shaanxi Province, CO Colombia, EC Ecuador, ID Indonesia, IN India Delhi, IR Iran, KG Kyrgyzstan, LA Lao PDR, LE Lebanon, MA Mauritius, ME Mexico City, MO Mongolia, SU Sudan, TA Tanzania, TJ Tajikistan, UG Uganda, UK Ukraine. Note: medicines in the public sector in BR, EC, ME, IN, UG, CO, LE and MA were dispensed free-of-charge to all patients in the outlets sampled so days’ wages are indicated as 0.

In the above analyses, medians across medicines have been used. Fig 1 is included to illustrate the situation for individual countries. It shows the availability and affordability of 30 days’ supply of lowest-priced generics of metformin 500mg (90 tablets) and 850mg (60 tablets) to treat diabetes. The right hand lower quadrant represents 80% or greater availability and 1 days’ wages or less to purchase treatment (or free-of-charge to all patients in the public sector outlets sampled). In the public sector, metformin was both available and affordable in only 4 countries (Mauritius, Lebanon, Iran and Colombia) of the 20 in the analysis. In the private sector, 4 countries (Lebanon, India (Delhi), Iran and Afghanistan) of the 21 in the analysis met the target.

## Discussion

Our analysis shows that low availability and/or poor affordability is resulting in few essential NCD medicines meeting WHO’s target in low- and middle-income countries. Lowest priced generics achieving the target in the public sector ranged from 15.2% in low-income countries to 36.0% in upper-middle income countries. In the private sector, the range was similar at 18.9% in low-income countries to 39.4% in upper-middle income countries. These unacceptably low levels are likely to be contributing to the high morbidity and premature mortality from NCDs seen in developing countries.

Despite its strengths, the WHO/HAI methodology has some limitations as outlined by Cameron et al.[8] (1) Medicine selection is limited to those with an MSH price and focuses on primary care, which may be reasons for the inclusion of few cancer medicines in surveys; (2) Alternate strengths, dosage forms or therapeutic alternatives are not taken into account; (3) Availability only refers to the day of data collection which may not reflect availability over time, although it does reflect the situation people experience when going to facilities; (4) The affordability metric does not include other healthcare costs (consultations, diagnostic tests etc.).

This is the first analysis of combined NCD medicine availability and affordability, and establishes a baseline for assessing future performance against the WHO target. In the era of Sustainable Development Goals, and countries working to achieve Universal Health Coverage, this baseline data shows that increased commitments are needed by governments and others to improve access to essential NCD medicines. Firstly, countries need to survey the availability, price and affordability of NCD medicines to identify target-gaps, then ascertain the determinants of low availability and/or poor affordability. From this, evidence-informed policies and interventions are needed that are fully implemented and enforced, and their impact monitored on a regular basis. While some of the interventions listed below have been subject to rigorous evaluation, the results may well depend on the country or environment when studied. This highlights the importance of continued monitoring and evaluation of policy interventions aimed at improving access to essential medicines.

The GAP and other documents [5, 19–21] outline a range of policy options to address low medicine availability, high prices and poor affordability. The most appropriate action depends on the national context but may include promoting competition through accelerated and lower-cost registration procedures for generics, efficient government procurement (national pooled procurement, buying lower-priced quality-assured generics, negotiating prices with suppliers), passing on low procurement prices where free medicines is not possible in the public sector, eliminating stock-outs through adequate forecasting, adequate and sustainable financing, efficient distribution, eliminating taxes and tariffs on essential medicines, regulating mark-ups in the supply chain (including importers, wholesalers, pharmacists), mandating prescribing by the medicine’s International Nonproprietary Name (INN), promoting generic substitution and incentivising the dispensing of lower-priced generics through regressive mark-ups or regressive dispensing fees (rather than the common practice of fixed percentage mark-ups that incentivises the dispensing of high priced products). Promoting the use of lower-priced generics to health professionals and the public is needed. Prerequisites to the acceptance and use of lower-priced generics include ensuring that products on the market are quality-assured, and the results of product quality testing are publicly available. Countries could also consider schemes to make high priced NCD medicines (such as inhalers and insulin) available in the private sector at low government procurement prices.

Whatever policies and interventions are used nationally, price transparency is vital as it empowers governments when procuring medicines, healthcare providers when prescribing, and, most importantly, patients when buying medicines. Governments should publish their tender prices, and ensure their citizens are easily able to compare patient prices. Likewise, governments should publicly report stock-outs.

As part of the plan to monitor the NCD indicators and targets, WHO and partner organisations should include availability, price and affordability of essential NCD medicines in all national surveys and monitoring work to be able to report on these critical indicators.

Momentum to improve access to medicines should build in response to the Sustainable Development Goals (SDGs), Universal Health Coverage (UHC) goals and other initiatives. Priority must be given to essential medicines to treat NCDs. It is hoped that these opportunities, along with the implementation of the WHO Global Plan of Action, will result in much needed improvement in access to NCD medicines in low- and middle-income countries.

This paper demonstrates that it is possible to measure access to NCD medicines in a robust way. If countries plan to implement the SDGs, UHC, and NCD goals they will need to measure and evaluate their performance. Many options exist for countries to meet these targets. Monitoring and evaluation of their actions will be crucial to learn from successes and failures that will occur.
